# The impact of polycystic ovary syndrome on the health-related quality of life: A systematic review and meta-analysis

**Published:** 2015-02

**Authors:** Fatemeh Bazarganipour, Seyed Abdolvahab Taghavi, Ali Montazeri, Fazlollah Ahmadi, Reza Chaman, Ahmad Khosravi

**Affiliations:** 1 *Hormozgan Fertility and Infertility Research Center, Hormozgan University of Medical Sciences, Bandarabbas, Iran.*; 2 *Mental Health Research Group, Health Metrics Research Center, Iranian Institute for Health Sciences Research, ACECR, Tehran, Iran.*; 3 *Department of Nursing, Tarbiat Modares University, Tehran, Iran.*; 4 *Department of Social Medicine, School of Medicine, Yasuj University of Medical Sciences, Yasuj, Iran.*; 5 *Center for Health Related Social and Behavioral Sciences Research, Shahroud University of Medical Sciences, Shahroud, Iran.*

**Keywords:** *Polycystic ovary syndrome*, *Review*, *Meta-analysis*, *Quality of life*

## Abstract

**Background:**

Polycystic ovary syndrome (PCOS) has been shown to cause a reduction in health-related quality of life (HRQOL). However, the relative degree of impairment in each domain differed among samples, and it was not clear which aspect of disease-specific HRQOL (modified polycystic ovary syndrome health-related quality of life questionnaire) was most negatively affected.

**Objective:**

To systematically review the effects of PCOS on specific domains of HRQOL.

**Materials and Methods:**

Literature search using search engine of database (PubMed, PsychInfo, CINAHL, CENTRAL, and Scopus) between 1998 to December 2013 yields 6 relevant publications. Pairs of raters used structural tools to analyze these articles, through critical appraisal and data extraction. The scores of each domain of polycystic ovarian syndrome questionnaire (PCOSQ) or modified version (MPCOSQ) of 1140 women with PCOS were used in meta-analysis.

**Results:**

The combine mean of emotional (4.40; 95% CI 3.77-5.04), infertility (4.13; 95% CI 3.81-4.45) and weight (3.88; 95% CI 2.33-5.42) dimensions were better, but menstruation (3.84; 95% CI 3.63-4.04) and hirsutism (3.81; 95% CI 3.26-4.35) domains were lower than the mean score of PCOSQ/MPCOSQ in related dimension.

**Conclusion:**

The meta-analysis showed that the most affected domains in specific HRQOL were hirsutism and menstruation. Based on these findings, we recommend healthcare providers to be made aware that HRQOL impairment of PCOS is mainly caused by their hirsutism and menstruation, which requires appropriate management.

## Introduction

A focus of medical research has traditionally been measurement of mortality and morbidity. As chronic diseases have become more prevalent, researchers have begun to recognize that these are not sufficient to capture the experience of disease (1). Patient-reported outcomes, including measurement of health-related quality of life (HRQOL), have emerged as important outcomes of interest. HRQOL is recognized as a subjective perception of wellbeing that is multidimensional and time and context dependent (2). Information obtained from HRQOL has numerous benefits. For example, assessment of treatment efficacy in clinical trials, identify needs and assign funds for patients by health care policymakers, monitor patient’s condition and make treatment decisions for busy clinicians. Polycystic ovary syndrome (PCOS) is the most frequent endocrine disease in reproductive age (3). The symptoms usually related with PCOS, including menstrual irregularities, hirsutism, anovulation and acne, can lead to a significant decrease in quality of life (QOL), mood disorders including depression, marital and social maladjustment and sexual dysfunction (4).

The PCOS health-related quality of life questionnaire (PCOSQ) is among well-developed disease specific instruments that was developed by Cronin *et al* (5). Cronin *et al* used semi-structured interviews, a health-practitioner survey and conducted a literature review to identify 182 items potentially relevant to women with PCOS. One hundred patients with PCOS reviewed the 182 items, decided which items were problems for them, and rated the importance of the items. The final PCOSQ includes 26 items and takes 10-15 min to self-administer. 

A factor analysis guided the categorization of the most important items into five areas or domains, namely concerns about emotion, hirsutism, weight, infertility, and menstruation. It has good reliability, but its validity showed controversial results due to absence of measuring acne (6-8). Thus, the PCOSQ was modified (MPCOSQ) by Barnard *et al* and four questions were added to the PCOSQ in order to evaluate issues associated to acne (9). Psychometric properties of the MPCOSQ in some population have been verified (9-11). 

Li *et al* performed a systematic review focusing specifically on general domain of HRQOL in women with PCOS. However, the relative degree of impairment in each domain differed among samples, and it was not clear which aspect of disease-specific HRQOL was most negatively affected. In this study, our goal was to systematically review the effects of PCOS on specific domains of HRQOL.

## Materials and methods

**Search strategy**

A database search was performed using PubMed, PsychInfo, CENTRAL, and Scopus which included papers written between 1998 to December 2013. The following keywords were used to search all databases for eligible studies: (Quality of life OR QOL OR HRQOL OR Health-related quality of life OR Wellbeing OR Satisfaction OR Health status OR questionnaire OR health status measurement OR quality of life questionnaire OR Psychometric OR Psychometrics OR Psychometry OR reliability OR validity OR Validation) AND (polycystic ovary syndrome OR polycystic ovaries OR PCOS OR polycystic ovarian syndrome). However, we preformed CINAHL database search between January1998 to December 2013 only with (Quality of life OR QOL OR HRQOL OR Health-related quality of life) AND (polycystic ovary syndrome OR polycystic ovaries OR PCOS OR polycystic ovarian syndrome) due to the block following the sanctions on Iran. 

**Exclusion and inclusion criteria**

Two investigators independently reviewed the title/abstracts of each reference identified by the search to identify relevant studies that have inclusion criteria. Differences were resolved by consensus. Studies were selected according to the following criteria: 1) the study presented original data, 2) the study included PCOS patients, 3) the study reported PCOSQ/ MPCOSQ subscales, 4) studies of any design that was written in English, 5) in the case of duplication with multiple articles publishing data on the same cohort, the most complete data set included, and 6) the studies reported outcomes as mean±SD.

**Quality assessment**

Two reviewers independently evaluated an assigned subset of articles using data extraction and quality appraisal tools. Two reviewers evaluated methodological quality independently using the strengthening the Reporting of Observational studies in Epidemiology (STROBE), which provides information on observational studies such as cohort studies, case control and cross sectional studies (12). After the independent evaluation, two reviewers met to discuss the article. Each specific item on the quality appraisal tool was openly discussed to reach consensus. This process identified whether disagreements were related to facts or adherence to the defined standards. Then, two investigators independently extracted the data from each selected study using a structured data extraction form. 

The following information was systematically extracted: 1) study design (e.g., randomized trial, cohort, cross-sectional, etc.); 2) the country where the study was performed; 3) characteristics of the patients: sample size, criteria used to identify patients with PCOS, Body Mass Index (BMI), Ferriman-Gallwey (FG) score, testosterone level; 4) name of used questionnaires; 5) and PCOSQ/ MPCOSQ subscale scores. For clinical trials and cohort studies, PCOSQ/ MPCOSQ scores at baseline were recorded. Authors of individual studies were contacted to obtain complementary data necessary to perform the meta-analysis and acquire uniform findings. Investigational review board approval was not required for this systematic review. 

**Statistical analysis**

The scores on questionnaires used to evaluate HRQOL in women with PCOS in each study were extracted as mean±SD and 95% confidence intervals (CI) were calculated for scores in all studies eligible for the meta-analysis and combined by using random-effects model (on basis of heterogeneity of included studies, we performed a random effects model). Statistical heterogeneity in the results of different studies was examined by ^2^ tests for significance. P<0.05 was considered statistically significant. To assess the extent of publication bias, a funnel plot may be used. Moreover, meta-regression analysis was carried out. The meta-analysis was conducted with Statistical software Stata 11 (StataCorp, College Station, TX, USA).

## Results

**Study characteristics**

**Figure 1:**
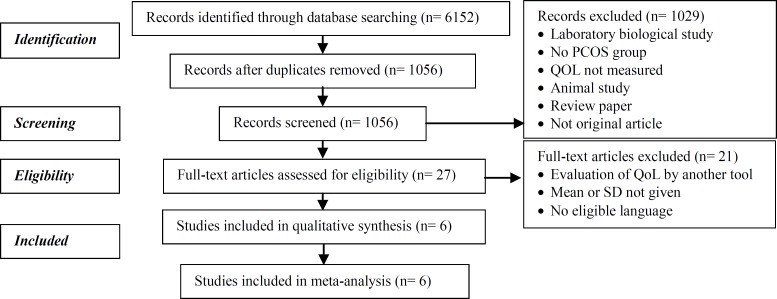
Flowchart shows the systematic review evidence

Figure 1 shows the flowchart of article selection according to the Preferred Reporting Items for Systematic reviews and Meta-Analyses (PRISMA) statement (13). The characteristics of the six studies selected for the current review are presented in table I. 

**Methodological quality**

The most common flaws observed in the identified articles related to quality appraisal were 1) not reporting study design in title, 2) inadequate sample size calculations/ justification, 3) absence report of missing data and how they were addressed, and 4) absence report of bounds of estimates such as confidence intervals (Table II). 

**Publication bias**

Because there were fewer than 10 included studies, potential publication bias was not assessed using a funnel plot or other corrective analytical methods (14).

**Meta-analysis**

A meta-analysis of 6 studies that used PCOSQ/ MPCOSQ was performed (6-7, 11, 15-17). Because only in one study used from MPCOSQ (additional acne dimension), we meta-analysis other five domain without acne (11). Included studies reported data on 1140 women with PCOS, and the number of subjects included in these studies ranged from 36-393. Data from each dimension of PCOSQ/ MPCOSQ were calculated in the meta-analysis (Table I).

**Emotional dimension**

**Figure 2:**
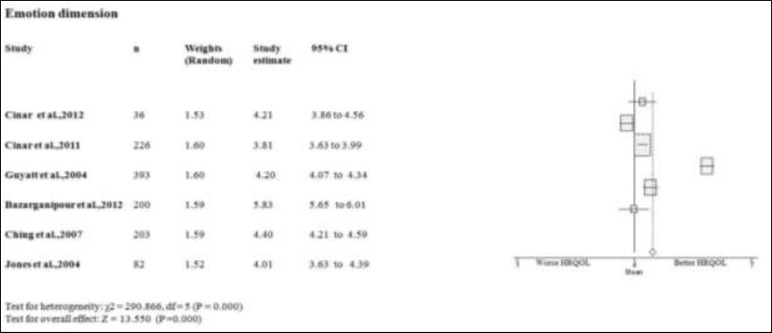
Forest plot of all included studies of HQROL in women with PCOS ( emotion domain)

**Figure 3:**
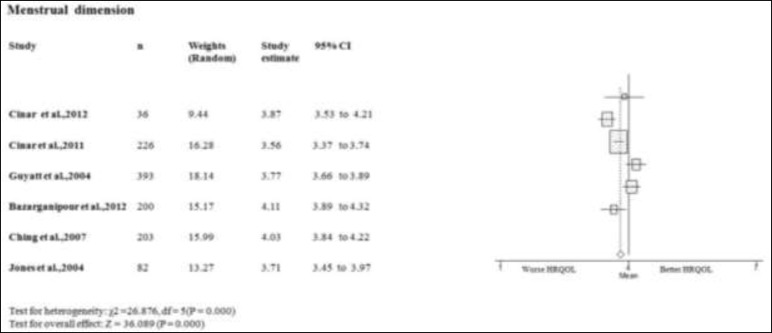
Forest plot of all included studies of HQROL in women with PCOS ( menstrual domain)

**Figure 4:**
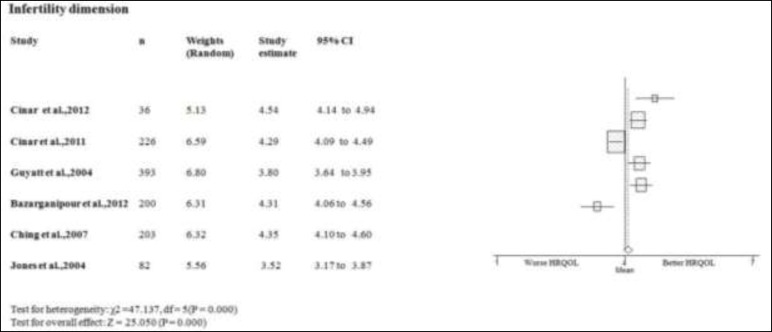
Forest plot of all included studies of HQROL in women with PCOS ( infertility domain)

**Figure 5:**
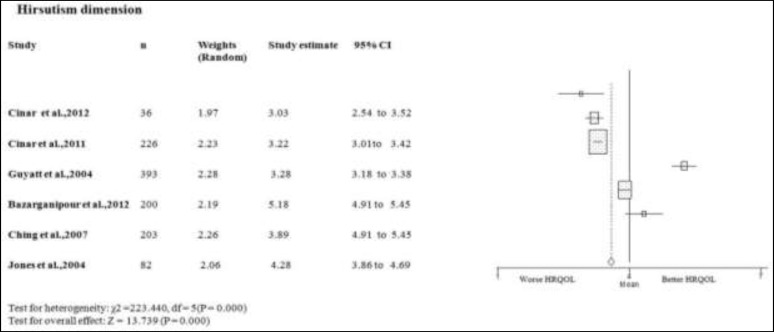
Forest plot of all included studies of HQROL in women with PCOS ( hirsutism domain)

**Figure 6:**
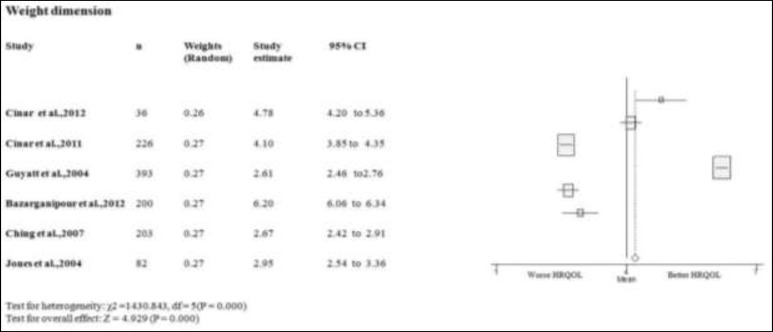
Forest plot of all included studies of HQROL in women with PCOS ( weight domain)

Five studies on the emotion domain of PCOS women scores better or equal than the mean of PCOSQ/ MPCOSQ (6, 7, 11, 15, 17). Only in one study this domain scores lower than mean of PCOSQ/ MPCOSQ (16). Also, the combine mean of emotion dimension is better than the mean score of PCOSQ/ MPCOSQ in this dimension (4.402; 95% CI 3.772-5.042). There was significant heterogeneity among studies (p<0.05) (Figure 2-6).

**Menstruation dimension**

Only in two studies on the menstruation domain of PCOS women scores better or equal than the mean of PCOSQ/ MPCOSQ (7, 17). In other four studies this domain scores lower than mean of PCOSQ/ MPCOSQ (6, 11, 15, 16). Also, the combine mean of menstruation dimension is lower than the mean score of PCOSQ/ MPCOSQ in this dimension (3.842; 95% CI 3.632-4.042). There was significant heterogeneity among studies (p<0.05) (Figure 3).

**Infertility dimension**

Four studies scores on the infertility domain of PCOS women better or equal than the mean of PCOSQ/ MPCOSQ (11, 15-17). In other two this domain scores lower than mean of PCOSQ/ MPCOSQ (6, 7). Also, the combine mean of infertility dimension is better than the mean score of PCOSQ/MPCOSQ in this dimension (4.132; 95% CI 3.812-4.452). There was significant heterogeneity among studies (p<0.05) (Figure 4).

**Hirsutism dimension**

Two studies scores on the hirsutism domain of PCOS women better or equal than the mean of PCOSQ/ MPCOSQ (7, 17). In other four this domain scores lower than mean of PCOSQ/ MPCOSQ (6, 11, 15-16). Also, the combine mean of hirsutism dimension is lower than the mean score of PCOSQ/ MPCOSQ in this dimension (3.812; 95% CI 3.262-4.352). There was significant heterogeneity among studies (p<0.05) (Figure 5).

**Weight dimension**

Three studies scores on the weight domain of PCOS women better or equal than the mean of PCOSQ/ MPCOSQ (7, 11, 15). In other three this domain scores lower than mean of PCOSQ/ MPCOSQ (6, 16, 17). Also, the combine mean of weight dimension is better than the mean score of PCOSQ/ MPCOSQ in this dimension (3.882; 95% CI 2.332-5.422). There was significant heterogeneity among studies (p<0.05) (Figure 6). The effect of heterogeneity was minimized by using a random effect model for all domains, as well as for meta-regression. We only have uniform data regarding BMI as confounder. 

Therefore, the meta-regression analysis investigated the association between the BMI and the five domains of HRQOL (Table III). The regression coefficients for all the domains were non-significant, with no evidence that the five HRQOL domains aren’t influenced by BMI.

**Table I:** Characteristics of the studies included in the systematic review

**PCOSQ/ MPCOSQ scores at baseline**	**Emotional**	4.2 ± 1.1	3.8 ± 1.4	4.2 ± 1.38	5.85 ± 1.33	4.40 ± 1.36	3.98 ± 1.8
	**Menstrual**	3.9 + 1.4	3.5 ± 1.5	3.77 ± 1.29	4.18 ± 1.70	4.07 ± 1.56	3.65 ± 1.48
	**Infertility**	4.7 ± 1.4	4.3 ± 1.6	3.78 ± 1.63	4.33 ± 1.95	4.38 ± 1.95	3.35 ± 1.88
	**Hirsutism**	2.9 ± 1.6	3.2 ± 1.6	3.28 ± 1.60	5.24 ± 2.21	3.89 ± 1.93	4.33 ± 2.04
	**Weight**	4.8 ± 1.8	4.1 ± 1.9	2.61 ± 1.58	6.20 ± 1.01	2.66 ± 1.80	2.94 ± 1.98
**Questionnaires **		PCOSQ, BDI, HADS, GHQ	PCOSQ, BDI, HADS, GHQ, STAI	PCOSQ	PCOSQ	PCOSQ, SF-36, GHQ-28, PIQ	PCOSQ, SF-36
**PCOS diagnostic criteria**		2003 Rotterdam criteria	2003 Rotterdam criteria	1990 NIH	2003 Rotterdam criteria	1990 NIH	N/A
**Testosterone (nmol/L)**		78.6 ± 31.3 (ng/dl)	N/A	N/A	N/A	N/A	40 (43.5)¥
**FG score**		8.7 ± 4.9	N/A	N/A	8.39 ± 4.28	N/A	46 (50)**
**BMI**		23.3 ± 4.8	24.7 ± 5.7	N/A	26.02 ± 4.03	33.6 ± 8.7	48 (54.2)*
**Location**		Turkey	Turkey	Canada, UK, USA	Iran	Australia	UK
**No. of PCOS group**		36	226	393	200	203	82
**Study type **		Prospective observational	Prospective Case-control	prospective randomized placebo-controlled blinded	observational cross-sectional	observational cross-sectional	observational cross-sectional
**Authors**		Cinar et al (2012)	Cinar et al (2011)	Guyatt et al (2004)	Bazarganipour et al (2012)	Ching et al (2007)	Jones et al (2004$)

* BMI>28

** Excessive body hair

¥ Testosterone >2

$ The scores of PCOSQ were transformed on a range from 0 (indicating worst health status) to 100 (best health status) for compare with the SF-36 in this study. We transformed to 0-6 again for comparison with another studies. Moreover, we add 1 to score of PCOSQ, because in another study range was1-7 but in this study was 0-6.

**Table II:** Quality of included studies

**Item/recommendation**		**Cinar et al** **2012**	**Cinar et al** **2011**	**Guyatt et al** 2004*	**Bazarganipour et al** **2012**	**Ching et al** **2007**	**Jones et al** **2004**
Title and abstract							
	Indicate study design		No	No	No	No	No
	Provide informative summary						
Introduction							
	Explain scientific background and rationale						
	State specific objectives/hypotheses						
Methods							
	Present key elements of study design						
	Describe setting, location, and relevant dates						
	Give eligibility criteria, selection/ follow-up methods						
	Give matching criteria	NA		NA	NA	NA	NA
	Define outcomes, exposures, and confounders						
	Give assessment methods for all variables						
	Describe efforts to assess potential sources of bias						
	Explain how the study size was arrived at	No	No	No		No	
	Explain how variables were handled in analyses						
	Describe all statistical methods						
	Describe methods to examine subgroups/ interaction	NA	NA	NA	NA	NA	NA
	Explain how missing data were addressed	NA	NA		NA	NA	NA
	Explain how loss to follow-up was addressed	NA	NA		NA	NA	NA
	Explain how matching was addressed	NA	No	NA	NA	NA	NA
	Describe any sensitivity analyses	NA	NA	NA	NA	NA	NA
Results							
	Report participant numbers at each study stage						
	Give reasons for nonparticipation at each stage	No	No		NA		No
	Consider using a flow diagram	No	No	No	No	No	No
	Give study population characteristics						
	Indicate % missing for all variables	NA	NA		NA	NA	NA
	Summarize follow-up time				NA	NA	NA
	Give unadjusted/adjusted estimates & precision						
	Give category bounds for categorized continuous variables	No	No		No	No	No
	Consider translating RR estimates to absolute risk	NA	NA	NA	NA	NA	NA
Discussion							
	Summarize key results						
	Discuss study limitations			No		No	
	Give a cautious overall interpretation of results						
	Discuss generalizability of study results						
Other information							
	Give source of funding and role of funders			No			No

NA: not applicable

**Table III:** Parameter estimates for meta-regression model

**Parameter **	**Domain of questionnaire**	**Coefficient ()**	**SE**	**p-value**	**95% CI**
BMI	Emotion	0.011	0.13	0.94	-0.58 to 0.60
	Menstruation	-0.29	0.04	0.56	-0.15 to 0.21
	Infertility	-0.006	-0.19	0.97	-0.08 to 0.08
	Hirsutism	-0.59	0.15	0.73	-0.60 to 0.72
	Weight	-0.22	0.16	0.31	-0.93 to 0.49

## Discussion

Since the early 1970s, interest in the concept of QOL has increased significantly, both in research and clinical practice. QOL has emerged as an important parameter for evaluating the quality and outcome of health care. This is especially the case for patients with chronic disorders like PCOS for whom QOL has become a critical outcome measure, since complete cure of disease is often unlikely. In this systematic review, we comprehensively evaluated the impact of PCOS upon women’s HRQOL. Our results indicate that the most significant affected domains of HRQOL in PCOS were hirsutism and menstruation. Approximately 60-70% of women with PCOS have hirsutism (18). 

From these numbers it is clear that this is an important issue for women with PCOS. Hirsutism is often cited by patients as being one of the most disturbing aspects of PCOS, causing marked psychological stress (19). Not only does the distress stem from the hair growth itself, but from the considerable amount of time and energy to try to keep the condition hide (20-22). Women with PCOS who experience hirsutism have often expressed that they feel “unfeminine”, “freakish”, “weird”, and “different” (23, 24). It is well known that the degree of hirsutism varies quite markedly with ethnicity. Although cosmetic and psychosexual consequences of hirsutism are recognized by some researchers to cause profound distress in affected persons. 

Sonino *et al* reported PCOS hirsute women have higher psychological distress and more interpersonal fears, suggesting a reduction of QOL (25). Fears reported in the hirsute women were categorized as “social phobia” or anxiety-evoking situations, such as meeting strangers, attending parties, shopping, and mixing at work. Schulman *et al* who investigated the psychopathological aspects of facial hirsutism, reported significantly elevated scores for negative mood and affect, which was unrelated to the degree of hirsutism and total testosterone (26). In a German PCOS cohort, the higher hirsutism score was associated with lower SF-36 scale include bodily pain, general health and physical sum scale, as well as with decreased sexual self-worth and sexual satisfaction (27). 

Kitzinger and Willmott reported that women with hirsutism described own as a “betrayal” (24). Hirsutism can be a very embarrassing characteristic for women, particularly on the face even if they shave. Based above mentioned, the skin manifestations of hyperandrogenism, specifically hirsutism, are strongly associated with both body dissatisfaction and depression. Patient complaints about skin problems should be sincerely and thoughtfully considered, not viewed as a trivial, cosmetic inconvenience. Most hirsute women spend considerable time and energy attempting to control facial hair, attention to appearance that in fact appears to facilitate better adjustment. 

This study brings into question: Are there differences between hirsutism in different cultures? HRQOL may vary from one population to another according to differences in cultural heritage, value systems, family structure, medical systems, values and norms related to illness-related communication, and other factors. As might be expected, cultural variables appear to mediate women’s responses to the different symptom dimensions. For example, relative to Austrian comparison samples, Brazilian women expressed significantly more concern about hirsutism, infertility, and menstrual irregularities; Moslem immigrant women expressed significantly more concern about infertility and menstrual irregularities (28). In cultures with expectations that women will have children are very strong, it is likely that the inability to do so will have a particularly negative impact on QOL. Due to the limited amount of research in this area; health care professionals need to pay attention to the psychosocial dimension of PCOS on an individual basis. Moreover, based on our findings, not only is excessive hair a concern, menstruation dimension has also posed as a difficulty.

The importance of menstruation issue in HRQOL for PCOS women can be discussed from several points. First, as major components of feminine role expectations, loss of regular menstruation may also cause or contribute to emotional distress in women with PCOS. It has been reported that not only visible characterizes of PCOS for example hirsutism but also the absence of their menstruation (amenorrhea) was negatively associated with fear about negative evaluation of people (29). This association might be related to reduced feeling of femininity (24). Second, menstrual irregularities can have important social consequences, especially in many Muslim countries. For example, the tenets of Islam decree that menstruating women cannot pray (30). 

If a woman is visibly not engaging in prayer for more than the expected four or five days per month, her whole household and social entourage is likely to be aware that she is experiencing menstrual irregularities (31). Moreover, Islamic texts forbid men from having sex with menstruating women as does Jewish and Zoroastrian faiths. Finally, menstrual irregularities are strongly related with infertility. However, some socio-cultural generalizations are possible: the social pressure to have a child shortly after marriage is strong in the some countries. For the infertile women, childlessness is an enormous psychological burden often associated with divorce, low social status and lowered self-perception because motherhood is perceived as an important part of female identity. In some cultures, motherhood is the only way for women to enhance status in their family and community. A study such as this is not without limitations. 

A systematic review of published studies is limited by the fact that it excludes unpublished data and this may result in publication bias, whereby studies with negative results may be less likely to have been published and included in the analysis. Another limitation of this review is the clinical heterogeneity of studies included. Heterogeneity may be introduced because of methodological and demographic difference among studies. Based on above mentioned, HRQOL may be different from one population to another according to different socio-cultural views. We used appropriate well-motivated inclusion criteria to maximize homogeneity and investigate potential source (BMI) of heterogeneity to assess its contribution to our findings.

Unfortunately, we did not have another characteristic of PCOS and could not perform a meta-regression for that part of the analysis. We identified some source of between study heterogeneity: the inclusion/ exclusion criteria, study design, patients’ demographics and disease severity. However, it should be noted that our goal was which aspect of domain of HRQOL were most affected not the magnitude of combine mean. Therefore, it’s not seemed our result affected by this limitation. Further, no gold standards exist to assess the study quality related to HRQOL. We used STROBE checklist for quality appraisal. Several points in the quality assessment of the research are important. First, the results of some studies for example Guyatt *et al* were presented in original article and not have some characterized included setting of patients and etc. Second, percentage of participation and missing data was not provided in some studies. However, we suppose that the prevalence of participation were 100% without missing data in these studies. Third, in some studies (internet survey), diagnosis of PCOS were based on patient statement not physician. It is supposed, the patient was informed of the diagnosis of PCOS based on physician diagnosis and this cannot be an important source of bias. Fourth, the STROBE checklist is critical article form not study. For example, in this checklist it’s necessary to define outcomes, exposures, and confounders. If this sentence isn’t included in the article, does not mean that the work is not done. Moreover, in almost all studies for their design, there is selection bias because the most of sampling was done in gynecological clinics. Also, patients referred to gynecology clinics may have different socio-cultural and psychological level compare to population. 

It is possible to recommend that most of these patients have menstrual irregularities and infertility because the patients with other complaints (not obesity and acne) are often go to other special clinics. But, the demographic findings of studies have shown sufficient number of patients with these complaints that allow evaluating QOL in them. Finally, although Guyatt *et al* study was a RCT, but the main objective of this study was to evaluate the psychometrics characteristics of questionnaire. We used the baseline scores of questionnaire for present study. Because our goal was not effect assessment, the STEOBE checklist was used for quality appraisal regarding this study.

Our study also has several strengths. It is the first meta-analysis of studies on this topic. We comprehensively searched multiple electronic databases in addition to manual searching. Also, we contacted authors for data leading to additional results from four studies. The significance of present study is two-fold. First, it provides evidence of the magnitude of impairment in diminished related dimension of HRQOL in PCOS. Thus, this study provides strong evidence that HRQOL is considerably impaired both in the hirsutism and menstruation domains in PCOS. Second, policymakers may be unfamiliar with this devastating disease in relation with HRQOL. This information, which shows significant impairment in the two mentioned domains of HRQOL in PCOS patients, provides important facts that physicians can use to assign funds for patients who have this disturbing disorder.

This further emphasizes both the importance of hirsutism and menstruation as a contributor to impaired HRQOL in PCOS and the treatment of hyperandrogenism and menstrual irregularities in improving psychological function in PCOS. Several lessons can be gleaned from this review and should be considered in future studies. Appropriate designed and well-conducted studies on the HRQOL of women with PCOS should be conducted in different countries. Future studies should include larger sample sizes and more generally representative sample. Moreover, prospective study design is needed to elucidate the role of demographic and PCOS symptoms in HRQOL in these patients. 

## Conclusion

In conclusion, this study provides evidence that HRQOL is impaired in patients with PCOS mostly by hirsutism and menstruation. This finding should now serve as our call to action to identify targets and implement interventions that have the ability to improve the HRQOL of those living with this devastating disease.
